# Can irrelevant but salient visual cues compensate for the age-related decline in cognitive conflict resolution?—An ERP study

**DOI:** 10.1371/journal.pone.0233496

**Published:** 2020-05-20

**Authors:** Boglárka Nagy, István Czigler, Domonkos File, Zsófia Anna Gaál

**Affiliations:** 1 Institute of Cognitive Neuroscience and Psychology, Research Centre for Natural Sciences, Budapest, Hungary; 2 Doctoral School of Psychology (Cognitive Science), Budapest University of Technology and Economics, Budapest, Hungary; 3 Institute of Psychology, Eötvös Loránd University, Budapest, Hungary; Universita degli Studi di Roma La Sapienza, ITALY

## Abstract

We studied a Posner-type gaze-cued version of a Simon task to characterize age-related changes in visuospatial attention and inhibitory control. Earlier results had indicated that the direction of gaze is a strong social cue that speeds response times; so we wondered whether, as a task-irrelevant stimulus, it could compensate for age-related impairment of inhibitory processes in the elderly. Our results assessed the Simon effect by: reaction time, error rate, the P3 component and the lateralized readiness potential (LRP). We found that the Simon effect was larger in the older group confirming an increased sensitivity to interference and also suggesting a decreased inhibitory control in older adults. LRP results showed that aging and stimulus-response incongruency delayed the selection of the responses–indexed by longer s-LRP latency data–, and also decreased the efficiency of motor inhibition in the Simon task–the s-LRP amplitude of both wrong- and correct-side activation was larger in older adults, and the latency difference of these two components was longer in this age-group. Also a larger N2pc amplitude in the congruent, compared to incongruent gaze condition, showed an increased visuospatial attention when the gaze-cueing drew attention to the target stimulus. This gaze-cueing could not be ignored and hence it modified task processing in the older age group, which was evident in the incongruent Simon condition where the congruent gaze increased older adults’ reaction time and their error rate; but there was no difference observed in the congruent Simon condition. Since the anticipated facilitation of reaction times did not occur, we suggest that general slowing and decreased inhibitory functions in the elderly caused the social cue not to be a supporting stimulus but rather to be a further burden on their cognitive processing.

## Introduction

Inhibitory processes are thought to play a central role in the understanding of how distractibility and interference sensitivity in older adults result in age-related changes in several cognitive domains–as first suggested by Hasher and Zacks [1]. Here, we studied these phenomena in a visuospatial conflict monitoring task with the aim of developing an appropriate method for compensating age-related impairment in the elderly. We looked for a simple method with a robust effect and identified gaze direction cueing as fulfilling these requirements, because it is not only a readily accessible ecologically valid stimulus but also has a strong attentional influence.

Key advantages of gaze cueing are its strong biological background and the ability to automatically direct visual attention towards the gaze direction (see a review by [2]). The specialization of the neural system in the process of human beings becoming an expert in facial recognition begins during the first days of life. With faces, we are very sensitive particularly to the eyes and processing their gaze, which develops fast from birth (reviewed by [3]) as a five-month-old infant can already distinguish a five-degree change in a person’s gaze [4]. The superior temporal sulcus is responsible for gaze perception [5], as this area accommodates a large set of information, such as understanding emotions and mental states in others; and hence it has an important role in the development of social cognition, in particular for communication, learning and attachment [6].

In our experiment we used the attention orienting role of gaze. Orienting is well-studied with different versions of the spatial cueing paradigm [7] with the result that targets on the stimulated location were processed more efficiently than targets on the opposite side [8,9]. In a gaze cueing version of this paradigm participants fixate on the centre of the screen and make a quick response when the target appears either to the left or right of the fixation point. In the centre of the screen a face as a cue is presented looking either to the left or to the right before the target stimulus appears. Importantly, the gaze orientation does not predict the target location, therefore in principle it is an uninformative cue. However, the reaction times were found to be faster in congruent compared to incongruent trials either with line-drawn faces [10] or photographs [11]. Hietanen and his colleagues [12,13] also showed that the attention orienting effect of the gaze and the traditional arrow cues were based on different cortical networks and mechanisms. As for the terminology: in congruent trials the target is presented at the gazed-at-side; in incongruent trials this is the opposite side, while in neutral trials the face gazes ahead. The faces in the present study were task-irrelevant stimuli, and we hypothesized that young adults would be able to ignore them successfully; while older adults would not, and these stimuli would interfere with their performance of the original task. We sought to find out whether or not the non-informative gaze cue was strong enough to modify such a demanding response inhibition phenomenon like the Simon effect.

One possible consequence of the age-related decline and impairment in cognitive control processes is a decreased efficiency in monitoring and resolving cognitive conflicts. Egner [14] emphasized that the conflict monitoring is not domain-general (conflict monitoring theory, [15]) but can be divided into perceptual and motor conflict processes which can be detected and observed with commonly used cognitive conflict tasks such as the Simon task [16]. The Simon task is a specific stimulus-response conflict task (spatial conflict task) in which participants have to give a spatial response (pressing the left or right button) to the value taken by spatially lateralized stimuli on a non-spatial dimension (e.g., letters–as was in our version). The task-irrelevant location of the target stimuli (e.g., left side of the visual field) and the response side can be the same, constituting the congruent condition (e.g., left hand response); or different, constituting the incongruent condition (e.g., right hand response). The Simon effect indicates that reaction times for incongruent trials are slower compared to congruent trials in which the spatial dimension of the target and the response are matched [16]. The Simon effect was found to be smaller when the pre-cue validly predicted the target location than at the uncued [17] or invalidly cued location [18].

As earlier studies have revealed age-related decline of cognitive control in the Simon task–mainly by the decreased efficiency of motor inhibition and response selection [19,20]–and, as the attentional resources and orientation influence the efficiency of cognitive conflict processes and resolution, we were interested to determine if age-related impaired cognitive control could have an impact on conflict resolution. So we decided to combine a visuospatial central gaze cueing task [21] with the Simon task in order to examine the magnitude and temporal course of their interaction by measuring reaction time (RT), error rate and the event-related potential components (ERPs)–in particular the P3b, N2pc and the lateralized readiness potential (LRP) components.

The P3b is a positively deflected ERP component with 250–500 ms latency after stimulus onset and a parietal maximum. In oddball paradigms, the P3b amplitude changes with the temporal probability of the stimulus, task demands and attentional resource allocation [22]. This component is related not only to context updating [23], but is also considered to be a component linking perceptual processing with response preparation [24]. Moreover, research into cognitive conflict has found that the P3b amplitude is larger for congruent trials [25,26]. Also, aging studies in various paradigms have revealed that older people have a decreased amplitude, a delayed latency and a more frontal distribution of the P3b component when compared with young adults [27]. And, in conflict task studies, older adults exhibit an increased modulation of the P3 amplitude–evidenced by a larger decrease for incongruent trials than congruent ones–and hence, the P3 amplitude is smaller for incongruent trials. This finding by Korsch et al. [28] might have been due to the higher task demands and the larger cognitive resources required.

Lateralized readiness potential (LRP) is a movement-related ERP component in connection with response preparation which originates from lateralized activation at the motor cortex. More precisely, LRP is the negative contralateral-minus-ipsilateral difference wave of the evoked potentials recorded from the C3 and C4 electrode sites (or a bit more lateral and anterior to them, from C3’ and C4’) with respect to the responding hand [29]. Its onset and maximal negative difference peak can be detected as a stimulus-locked LRP (s-LRP) which identifies that point when motor activity in the brain begins to favour making one of the two possible responses or as response-locked LRP (r-LRP) which indicates the activation of motor programming processes which are required for the given response execution [30]. Hence, response interference (the stimulus-response spatial relationship in the Simon task) can be tested with this component. When response inhibition is not efficient, higher r-LRP amplitude can be measured than in cases of successfully inhibited responses [31]. This difference can be seen in aging studies where larger r-LRP amplitude can be measured in older adults showing weaker response inhibition [30].

To control visuospatial attention and the influence of the task-irrelevant faces, we evaluated the N2pc component, which is a negative potential contralateral to the side of the target stimulus, and reaches its maximum at PO7, PO8 electrodes between 180–350 ms after stimulus onset [32]. This component has been extensively studied in visual discrimination tasks and is thought to correlate with the visual discrimination of the target stimulus [32,33]. Van der Lubbe and Verleger [19] did not find any aging effects on N2pc (PCN) amplitude in a Simon task, but the latency was longer in the older compared to younger group. In interpreting this result they considered that although the age-groups did not differ in their efficiency of the target discrimination, this process started later in older adults, which indicated a slowing of their attentional processing. In the present study we used the N2pc to show age-related differences in visuospatial attention and also to reveal the effect of the task-irrelevant gaze-cueing in examining attentional processing.

**Fig 1:**
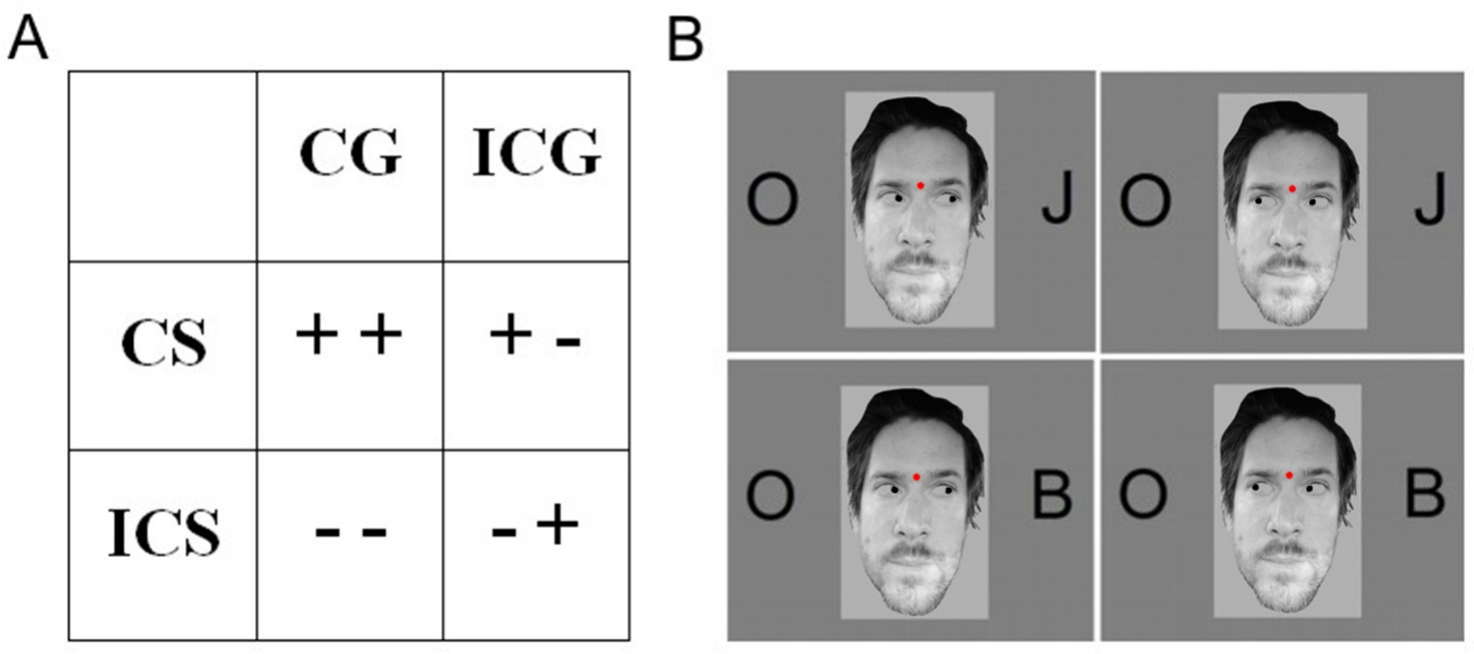
Different combinations of simon and putative gaze effects. (A) Different levels of correct response facilitation (+: facilitation,—: inhibition) for different combinations (trials) of Simon and Gaze conditions (CS: congruent Simon, ICS: incongruent Simon, CG: congruent Gaze, ICG: incongruent Gaze). (B) Stimuli of the four conditions (example): CS-CG on the top left, CS-ICG on the top right, ICS-CG on the bottom left, ICS-ICG on the bottom right.

Our hypothesis was that the Simon effect would not only be observed in the RT, P3b and LRP components, but also would be higher in the elderly. If the gaze cues were successfully ignored then they would not change the Simon effect. However, we assumed that older adults, as distinct from young adults, would not be able to ignore the effect of the task-irrelevant cues, and hence the correct hand response in this age group would be influenced by the different combinations of Simon and gaze conditions (Fig 1). As earlier results [12,13] showed that a centrally presented gaze automatically shifts visuospatial attention and facilitates target processing at the cued site, we hypothesized that in the congruent gaze condition attention is drawn to the target which results in 1) attention being drawn to the response side in congruent gaze–congruent Simon condition; and 2) attention being drawn to the opposite side in congruent gaze–incongruent Simon condition. We assumed that in comparing the incongruent gaze conditions for the former (1) a facilitatory effect and for the latter (2) an inhibitory effect will be observed. Consequently, the reaction time will either decrease (1) or increase (2), demonstrating that the elderly, with some external help, could overcome the disadvantages of deteriorated inhibitory processes.

## Materials and methods

### Participants

There were two age-groups. In the young group there were 24 participants (mean age: 22.0, SD = 2.3, range 18–27 years; 12 female). In the older group there were 21 participants (mean age: 68.1, SD = 3.25, range: 67–74 years; 10 female). Due to technical reasons an older participant’s data was omitted. Full scale Wechsler IQ (measured by the Hungarian version of WAIS-IV, [34]) was conducted in both age-groups in order to rule out dementia-related differences: IQ(young) = 107.7±16.6; IQ(old) = 128.9±15.3. Every participant was right-handed, had normal or corrected-to-normal vision, and had no history of any kind of neurological or psychiatric disorder. All participants were paid for their contribution.

The protocol was approved by the Joint Psychological Research Ethics Committee (EPKEB, Hungary) and a written informed consent was obtained from all participants.

### Procedure

**Fig 2:**
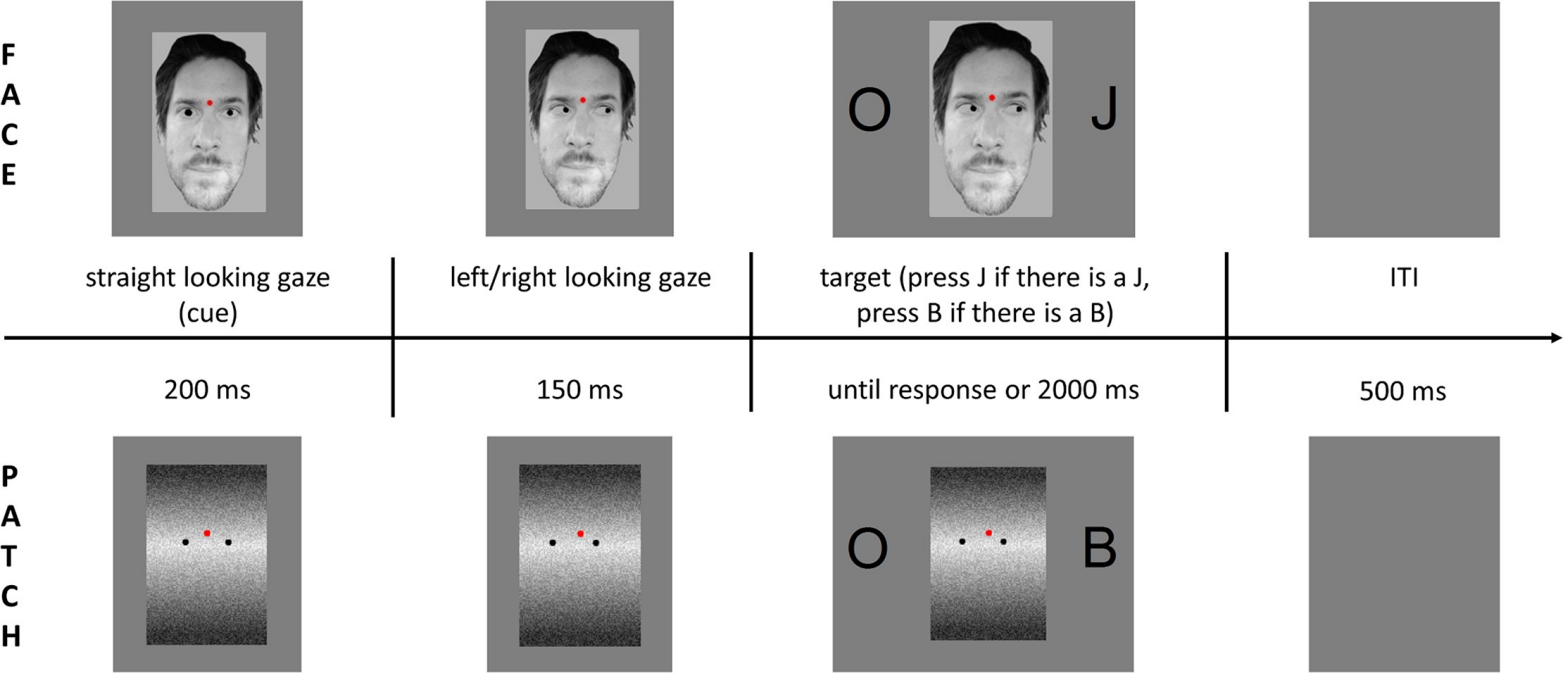
Illustration of the sequence and timing of stimuli in the face cued trials (top row) and the patch cued trials (bottom row). ITI is for intertrial interval.

Each participant was seated in a comfortable chair in a sound-attenuated and electrically shielded chamber. The experimental stimuli were presented with MATLAB R2016b (The MathWorks, Inc.) using a 19 inch CRT monitor (LG Flatron F920B, 75 Hz refresh rate) from 1.2 m distance. The experimental design is shown in Fig 2. Trials started either with a straight-looking face or a patch (task-irrelevant/distracting stimulus), the patch being a control for the gazing face, on a grey background, and with a red fixation dot which was presented for 200 ms. Next, a left- or right-looking gaze appeared for 150 ms (task-irrelevant gaze cue) followed by two symmetrically positioned black letters on the left and right side of the gazing face or patch stimulus. The letters were presented until the participants responded, or for a maximum 2,000 ms (timeout). One of the letters was always O and the other letter was either B or J (target letters which are the Hungarian abbreviations for left—*b**al* and right—*j**obb*). The size of the letters was 1.4°× 1.3° visual angle from the 120 cm viewing distance. The direction of the gaze (left or right), the identity of the letter (B or J), and the side of the target letter (left or right) varied randomly, and with equal probability; however, two consecutive trials had to differ at least in one dimension. After a response, a blank grey background was presented for 500 ms (inter-trial interval). The session started with a practise block with 50 patch trials. Half of the participants started with the face condition and the other half with the patch condition. Both the face and patch conditions contained 400 trials, in 8 blocks of 50 trials. In the face condition, a different face stimulus was shown in every trial, resulting in 50 different faces being used in one block and all of these 50 faces were reused in all the face blocks.

Face stimuli for our experiment were collected from online free sources, and were frontal, male, neutral or slightly happy faces. Corel Photo-Paint X3 was used to manipulate the eyes in these images to look either straight ahead, or to the left or to the right. In the patch condition the patch stimulus was created by using 55 squares of different sizes (3.7×6.7 px– 19×53 px) and shades of grey (rgba(36, 36, 36, 1)–rgba (249, 249, 249, 1)) which filled a predefined rectangle and were placed in a random order but in such a way as to not look face-like. Additionally, we applied Gaussian blur to each square and set the luminance similar to the face images. Two black dots were placed at the same position as the eyes and left/right-looking gaze was imitated by moving these two dots correspondingly. The patch stimuli (straight/left/right-looking) were generated beforehand and they were the same during the whole experiment for each participant. Both face and patch images were seen under 3.3° × 4.1° visual angle and a red dot fixation point was placed between the eyebrows on the face images and in the same position on the patch image.

### Task

Participants had to perform a modified visual Simon task with task-irrelevant gaze cueing (i.e. a two-alternative forced-choice task). They were instructed to fixate on the central red dot and not to look away from it, as well as to respond as fast and accurately as possible.

Participants were instructed to press the B button with their left hand on a prepared keyboard when letter B was presented and to press the J button with their right hand when the letter J was presented. Therefore, the side of the target stimulus and the required responding hand could correspond (congruent Simon condition, CS) either when B was on the left or J was on the right side, or alternatively, the incongruent Simon condition (ICS) was apparent either when B was on the right or J was on the left side. Moreover, the task-irrelevant gaze could be directed either to the target letter as congruent gaze cueing (CG) or to the opposite side as incongruent gaze cueing (ICG)–thus congruence of gaze cueing refers to the stimulus position. Feedback was given after each block, as the average reaction time and the number of errors. In summary, the four experimental conditions with face and patch stimuli were: congruent Simon–congruent gaze cueing (CS-CG); congruent Simon–incongruent gaze cueing (CS-ICG); incongruent Simon–congruent gaze cueing (ICS-CG); incongruent Simon–incongruent gaze cueing (ICS-ICG).

### ERP recording

EEG was recorded with a NeuroScan 4.5 recording system (NeuroScan SynAmps2 amplifier, USA, Brain Products EasyCap, Ag/AgCl electrodes, DC-200 Hz, sampling rate: 500 Hz). We used 28 locations in accordance with the extended 10–20 system: F7, F3, Fz, F4, F8, FC3, FC4, T7, C3, Cz, C4, T8, CP5, CP6, P7, P3, Pz, P4, P8, PO7, PO3, POz, PO4, PO8, O1, Oz, O2, with AFz as ground and the reference on the tip of the nose. Vertical eye movements (VEOG) were recorded from electrodes above the left eye (AF7) and below the left eye, and horizontal eye movements (HEOG) were recorded from electrodes placed at the outer canthi of either eye. The impedance of the electrodes was kept below 10 kΩ.

### ERP data analysis

Offline EEG processing was performed in MATLAB environment (The Mathworks, Inc.) and started with a non-causal Kaiser-windowed Finite Impulse Response filter (low pass filter parameters: 30 Hz cut off frequency, beta of 12.2653, a transition bandwidth of 10 Hz; high pass filter parameters: 0.1 Hz cut off frequency, beta of 5.6533, a transition bandwidth of 0.2 Hz). Independent Component Analysis (ICA) was applied on our filtered EEG data in order to reject eye-movement artifacts (blinking, looking aside) which was performed with the EEGLAB toolbox [35].

Segmentation was performed for stimulus-locked ERP components from -450 to 800 ms (N2pc, s-LRP, P3b) relative to letter (target) onset and for the response-locked component (r-LRP) from -400 to 200 ms in young, and -500 to 200 ms in older adults relative to the response. The baseline was -450 to -350 ms (-100 to 0 ms before the cue) for the N2pc, s-LRP and P3b components before the target stimulus appeared; and for the r-LRP -400 to -300 ms in young and -500 to -400 ms in older adults relative to the response. Epochs were rejected from averaging if they had a larger than 100 μV voltage change between the minimum and maximum of the epoch.

To obtain the s-LRP and r-LRP we calculated the symmetrical electrode pair differences for C3-C4, contra-minus-ipsilateral to the responding hand, and to obtain the N2pc we calculated the symmetrical electrode pair difference for P7-P8, contra-minus-ipsilateral to the presentation side of target stimulus. We calculated the LRP components separately from the average of the C3-C4 differences in the left and right handed responses, and then we took the grand average of the two-sided averages. And similarly, for the N2pc we calculated the average of P7-P8 differences of the left- and right-sided target presentation for all the conditions, and finally we took the grand average again of the two-sided averages.

### Data analysis

We measured reaction times (RT) locked to the appearance of the target stimuli and calculated error rates. Trials with incorrect responses, or with reaction times that were quicker than 150 ms, or slower than 1,500 ms, were excluded from further analysis. Median RTs of all correctly responded trials were determined in each condition for each participant in order to balance out intraindividual variability which tends to be higher in older adults [36,37].

ERP components were defined by searching for the largest positive (s-LRP early positivity in ICS conditions, P3b) or negative (N2pc, s-LRP, r-LRP) peak within the time windows as specified in Table 1. The amplitudes were calculated as the mean amplitude within ± 10 ms around peak latency for each participant. Onset latency of the negative deflection of s-LRP in CS conditions, of the positive deflection of s-LRP in ICS conditions and of the r-LRP were defined as the point when 30% of the maximal amplitude was reached (peak proportion method, [38,39]). Based on Gratton et al. [40] we analysed the s-LRP to measure wrong-side activation, and both the s-LRP and r-LRP were used to quantify the other aspects of response execution.

**Table 1:** Channels and time windows for ERP components’ amplitudes and latencies.

ERP component	Channels	Time window
**P3b**	Pz	young: 300–600 ms
		older: 300–700 ms
**N2pc**	P7-P8 difference	young: 200–300 ms
		older: 200–350 ms
**s-LRP early positivity**	C3-C4 difference	young: 200–300 ms
		older: 200–350 ms
**s-LRP**	C3-C4 difference	young: 200–500 ms
		older: 200–600 ms
**r-LRP**	C3-C4 difference	-200-0 ms

Time windows of P3b, N2pc, and s-LRP components are defined relative to the target stimulus, and the time window of the r-LRP component is defined relative to the response.

Statistical analyses were performed with Statistica 13 (TIBCO Software Inc.). Repeated measures of ANOVAs with *Age* (young/older) as between-subject factor were made. The within-subject factors were: *Simon* (CS/ICS); *Gaze* (CG/ICG); and *Cue* (face/patch) in both RT and ERP analyses. We used the factor *Letter* (J/B) only in RT analyses. The effect size was calculated as Cohen’s d for t-tests and as partial eta square (η_p_^2^) for ANOVAs. Post hoc analysis was performed by using the Tukey HSD test. T-tests were carried out for comparing means or detecting significant differences from the baseline.

## Results

### Behavioural data

**Fig 3:**
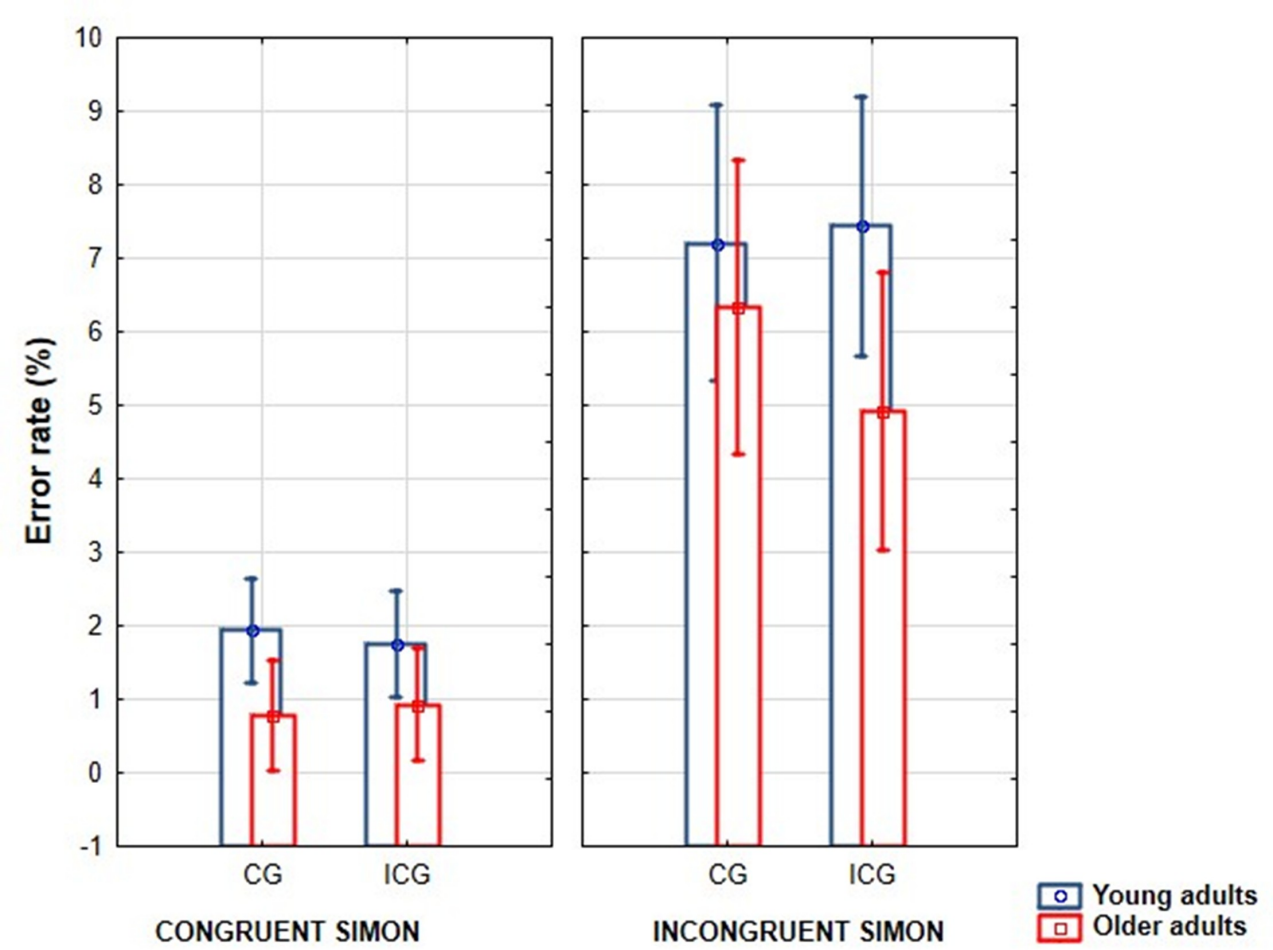
Error rates (%) in the congruent and incongruent simon tasks when the gaze was congruent (CG) and incongruent (ICG) in the young and older age groups. (Vertical bars denote 95% confidence intervals).

Fig 3 shows that older participants made significantly less errors than young ones in the modified visual Simon task (*Age* main effect: F(1,43) = 8.12, p = 0.007, η_p_^2^ = 0.16; M±SD (error%-older) = 3.2±1.6; M±SD (error%-young) = 4.6±1.5). All participants made more errors in the incongruent compared to the congruent Simon condition (*Simon* main effect: F(1,43) = 136.56, p < 0.001, η_p_^2^ = 0.76). The post hoc test of the *Simon x Gaze x Age* interaction (F(1,43) = 6.22, p = 0.017, η_p_^2^ = 0.13) showed that the gaze influenced the error rate only in the older group, where more errors were made in the incongruent Simon condition when the gaze was congruent rather than incongruent (p = 0.025). Age groups differed only in the ICS-ICG condition where young participants made more errors than the older adults (p = 0.020).

**Fig 4:**
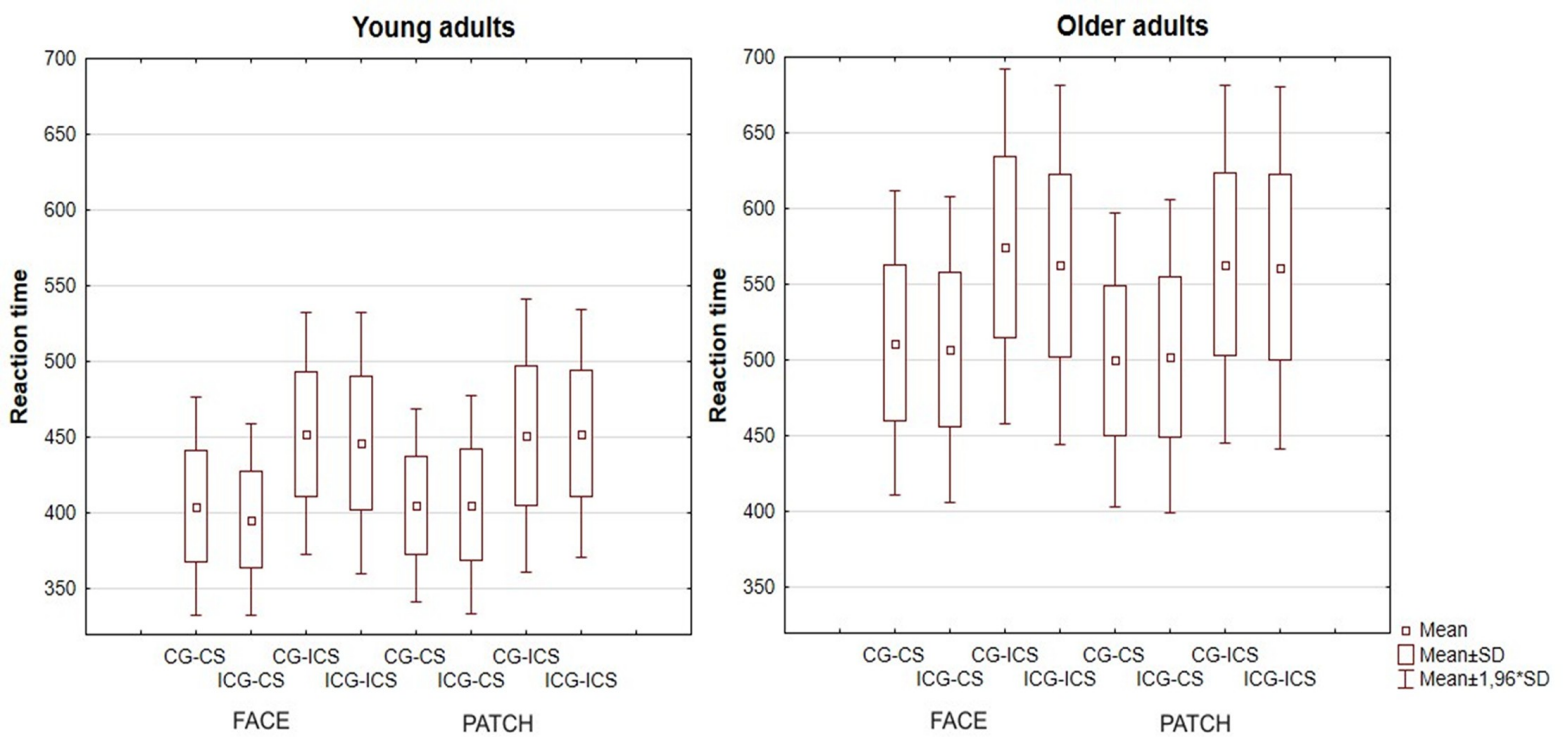
Reaction time data (mean and standard deviation) of young and older adults for face and patch cues in congruent gaze-congruent simon (CG-CS), incongruent gaze-congruent simon (ICG-CS), congruent gaze-incongruent simon (CG-ICS), and incongruent gaze-incongruent simon (ICG-ICS) conditions.

Reaction time data are shown in Fig 4. Young adults reacted faster than older adults (*Age* main effect: F(1,43) = 65.42, p < 0.001, η_p_^2^ = 0.60). Responses were significantly faster in the congruent compared to the incongruent Simon condition (*Simon* main effect: F(1,43) = 487.53, p < 0.001, η_p_^2^ = 0.92). According to the post hoc test, the *Age x Simon* interaction (F(1,43) = 6.25, p = 0.016, η_p_^2^ = 0.13) showed the same results as the main effects: every pair was significant, and the RT increased in the order of young CS (403±34 ms), young ICS (451±43 ms), older CS (505±50 ms), older ICS (566±60 ms). However, the gaze influenced this effect. The results showed that reaction times were slower in the congruent gaze condition compared to the incongruent one (*Gaze* main effect: F(1,43) = 22.07, p < 0.001, η_p_^2^ = 0.34, which was significant for the face condition (*Gaze* × *Cue* interaction: F(1,43) = 13.33, p < 0.001, η_p_^2^ = 0.24, post hoc p < 0.001), but not for the patch condition (p = 0.987). The *Simon* x *Gaze* x *Age* interaction (F(1,43) = 4.88, p = 0.033, η_p_^2^ = 0.10) showed that the gaze did not differentially affect RTs for Simon-congruent (p = 0.289) and Simon-incongruent (p = 0.911) trials in the young group, but did so in the older group: a slower RT was found in the incongruent Simon condition for the congruent compared to the incongruent gaze (p = 0.011), whereas there were no differences in the congruent Simon conditions (p = 0.999).

Examining in particular the Simon-effect, subtracting the RT of CS condition from ICS condition (young CG: 46.99±16.64 ms; ICG: 48.92±18.46 ms; older CG: 63.25±19.89 ms; ICG: 57.15±14.47 ms), we found an *Age* main effect (F(1,43) = 6.25, p = 0.016, η_p_^2^ = 0.13). *Age* x *Gaze* interaction F(1,43) = 4.88, p = 0.033, η_p_^2^ = 0.10) showed that older adults had significantly larger Simon-effect in congruently gaze cued condition compared to young adults (p *=* 0.015), but not in incongruent gaze cued condition (p = 0.402).

RTs were quicker for dominant hand responses, in this case right-handed responses, when the target letter was J, compared to subdominant hand responses, when target letter was B (*Letter* main effect: F(1,43) = 8.83, p = 0.005, η_p_^2^ = 0.17). This effect however, did not influence the main conclusions of the present paper and will not be discussed further here.

### Event-related potentials (ERPs)

**Fig 5:**
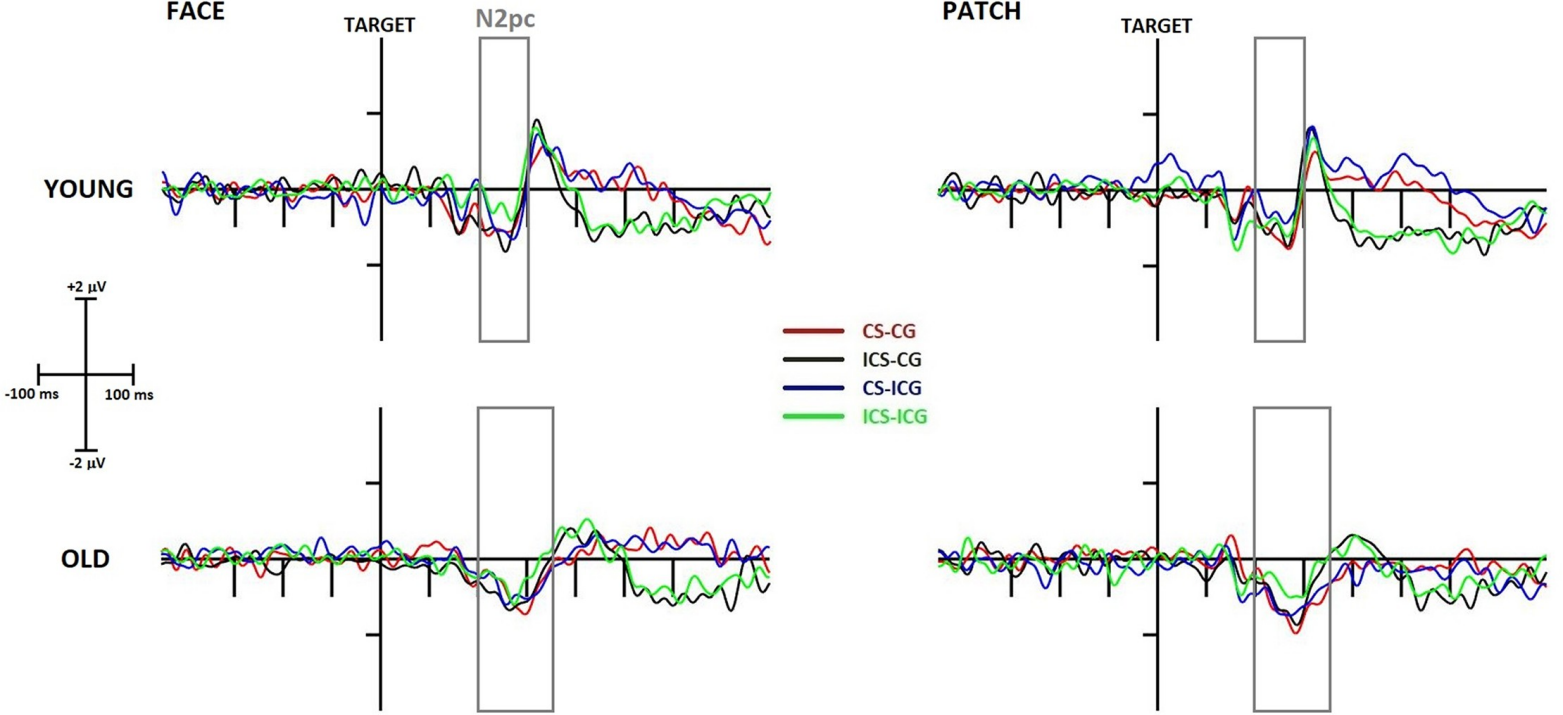
Target-locked N2pc component at the P7-P8 electrode difference (contra-minus-ipsilateral to the presentation side of the target stimulus) in young (top row) and older (bottom row) adults and in face (left side) and patch (right side) trials. The x-axis represents the epoch from -450 ms to 800 ms around the time-point of target presentation. CS-CG (congruent Simon, congruent gaze) waveforms are displayed in red, ICS (incongruent Simon)-CG waveforms in black, CS-ICG (incongruent gaze) waveforms in blue, and ICS-ICG waveforms in green. Grey rectangles represent the search window for maximal N2pc amplitude.

N2pc. The latency of the N2pc component (Fig 5) was longer in the older compared to young group (*Age* main effect: F(1,43) = 10.10, p = 0.003, η_p_^2^ = 0.19), but its amplitude did not show any age-related differences. However, we found a *Cue* main effect (F(1,43) = 4.72, p = 0.035, η_p_^2^ = 0.10), resulting from amplitudes being larger for patches than faces; and additionally a *Gaze* main effect (F(1,43) = 25.19, p < 0.001, η_p_^2^ = 0.37) resulting from increased amplitudes after congruent gazes compared to incongruent ones.

#### P3b

**Fig 6:**
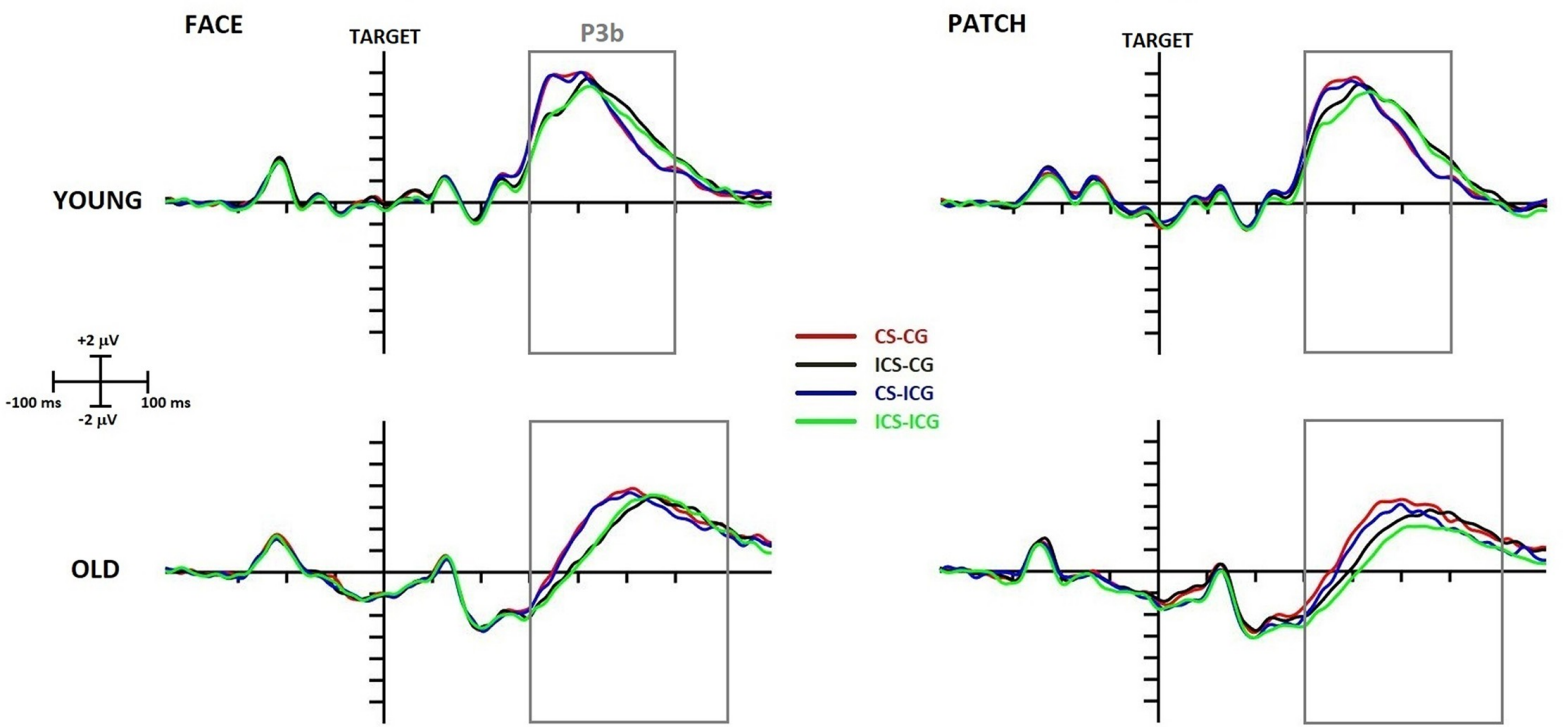
Target-locked P3b component at the Pz electrode site in young (top row) and older (bottom row) adults and in face (left side) and patch (right side) trials. The x-axis represents the epoch from -450 ms to 800 ms around the time-point of target presentation. CS-CG (congruent Simon, congruent gaze) waveforms are displayed in red, ICS (incongruent Simon)-CG waveforms in black, CS-ICG (incongruent gaze) waveforms in blue, and ICS-ICG waveforms in green. Grey rectangles represent the search window for maximal P3 amplitude.

As seen in Fig 6, older adults had longer P3b latencies than young adults (*Age* main effect: F(1,43) = 77.53, p < 0.001, η_p_^2^ = 0.64) and P3b latencies were longer in the incongruent than congruent Simon condition (*Simon* main effect: F(1,43) = 55.99, p < 0.001, η_p_^2^ = 0.57), but no other significant latency effect was observed. A larger amplitude was detected in young compared to older adults (*Age* main effect: F(1,43) = 8.08, p = 0.007, η_p_^2^ = 0.16), and amplitudes were larger in the congruent compared to the incongruent Simon condition (*Simon* main effect: F(1,43) = 8.56, p = 0.005, η_p_^2^ = 0.17). In face cued, as opposed to patch cued condition, there was a larger P3b component (*Cue* main effect: F(1,43) = 5.68, p = 0.022, η_p_^2^ = 0.12); however, the gaze did not influence this component.

#### Lateralized Readiness Potential (LRP)

**Fig 7:**
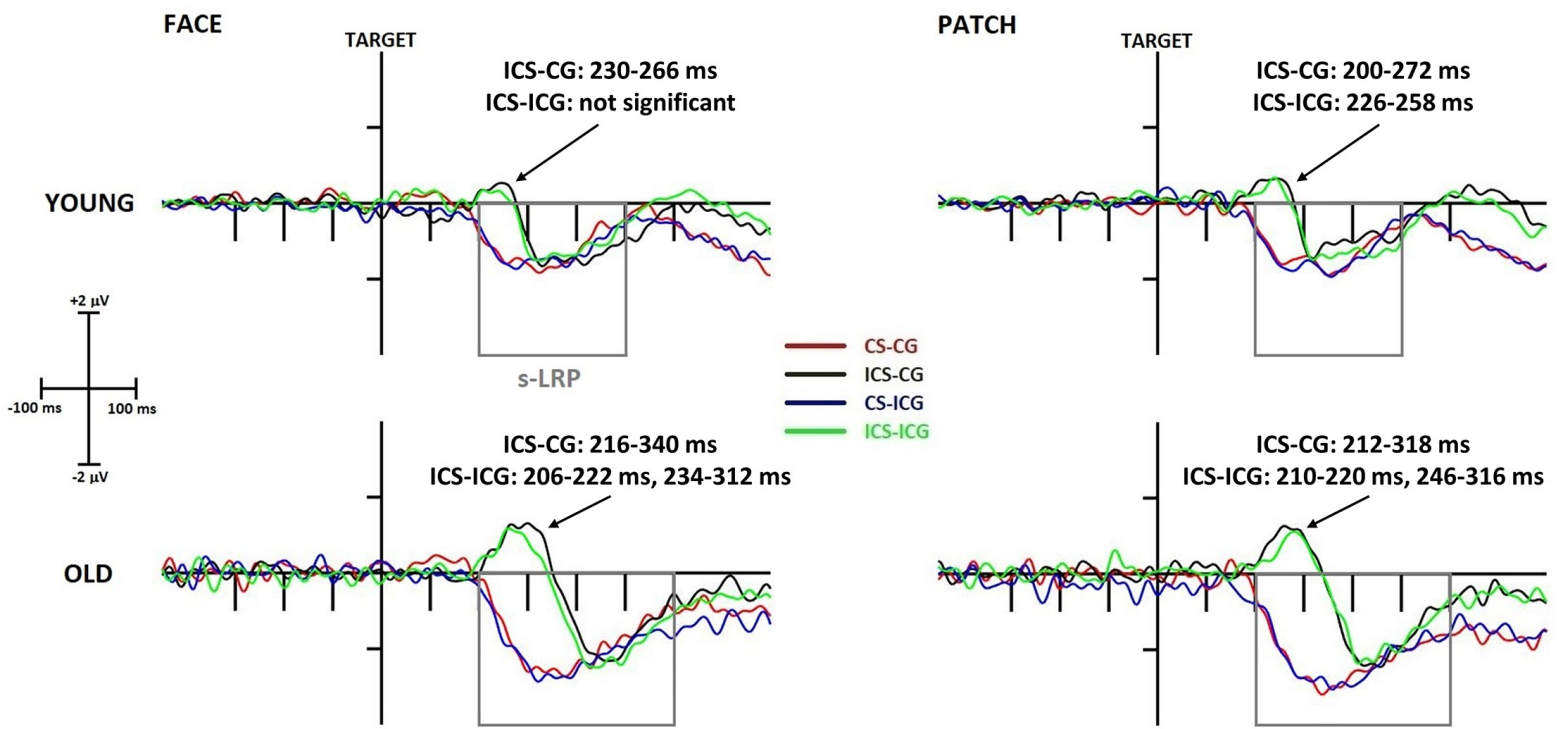
Stimulus-locked LRP component at the C3-C4 electrode difference (contra-minus-ipsilateral to the responding hand) in young (top panel) and older (bottom panel) adults and in face (left side) and patch (right side) trials. The x-axis represents the epoch from -450 ms to 800 ms around the time-point of target presentation. CS-CG (congruent Simon, congruent gaze) waveforms are displayed in red, ICS (incongruent Simon)-CG waveforms in black, CS-ICG (incongruent gaze) waveforms in blue, and ICS-ICG waveforms in green. The arrows show the positive dips of wrong side activation, and the latency ranges are indicated in which the t-test showed significant differences from the baseline in the 200–350 ms window. Grey rectangles represent the search window for maximal s-LRP amplitude in every subject.

*Stimulus-locked LRP*. As an initial analysis, we investigated whether any of the incompatible conditions evoked activity on the “wrong side” (for example left hand response for target letter J), one-sample t-tests were applied for the average of every data point’s amplitude of a post hoc given time window before the emergence of (correct response) s-LRP component in each condition. Therefore, we analysed if the averaged voltage value in the given time window is significantly larger (more positive) than 0 μV (baseline). Our criterion was to have 10 consecutive data points (20 ms) with significant (p<0.05) t-values. With one exception (ICS-ICG condition in young adults for face cues), we obtained significant deflection values (a positive dip) for young and older adults in the incongruent Simon conditions for both faces and patches, irrespective of gaze direction; but as expected, not for the congruent Simon conditions (see significant ranges in Fig 7).

This wrong-side activation, a positive dip, was larger in amplitude (*Age* main effect: F(1,43) = 10.11, p = 0.003, η_p_^2^ = 0.19) and longer in latency (*Age* main effect: F(1,43) = 52.43, p < 0.001, η_p_^2^ = 0.55) in the older rather than the young adults. The amplitude was influenced by the gaze with larger amplitudes being seen after congruent than after incongruent gazes (*Gaze* main effect: F(1,43) = 8.09, p = 0.007, η_p_^2^ = 0.14).

When we analysed the correct-side activation, the amplitude of the s-LRP was found to be larger in the older compared to the young age group (*Age* main effect: F(1,43) = 5.16, p = 0.028, η_p_^2^ = 0.11). The latency was longer in older than young adults (*Age* main effect: F(1,43) = 20.15, p < 0.001, η_p_^2^ = 0.32), and in the incongruent compared to the congruent Simon condition (*Simon* main effect: F(1,43) = 76.12, p < 0.001, η_p_^2^ = 0.64). We measured the onset latency of the negative s-LRP in the congruent Simon condition, and of the positive dip in the incongruent Simon condition to show the start of motor activation. We found prolonged latency in older compared to young adults in both cases (*Age* main effect in CS: F(1,43) = 4.16, p = 0.048, η_p_^2^ = 0.09; *Age* main effect in ICS: F(1,43) = 23.52, p < 0.001, η_p_^2^ = 0.35). We also calculated the latency difference between the peaks of the initial positive dip and the consecutive negative s-LRP component in the incongruent Simon condition. The results showed that older compared to young adults needed more time to overcome the initial wrong side activation (*Age* main effect: F(1,43) = 5.85, p = 0.020, η_p_^2^ = 0.12).

**Fig 8:**
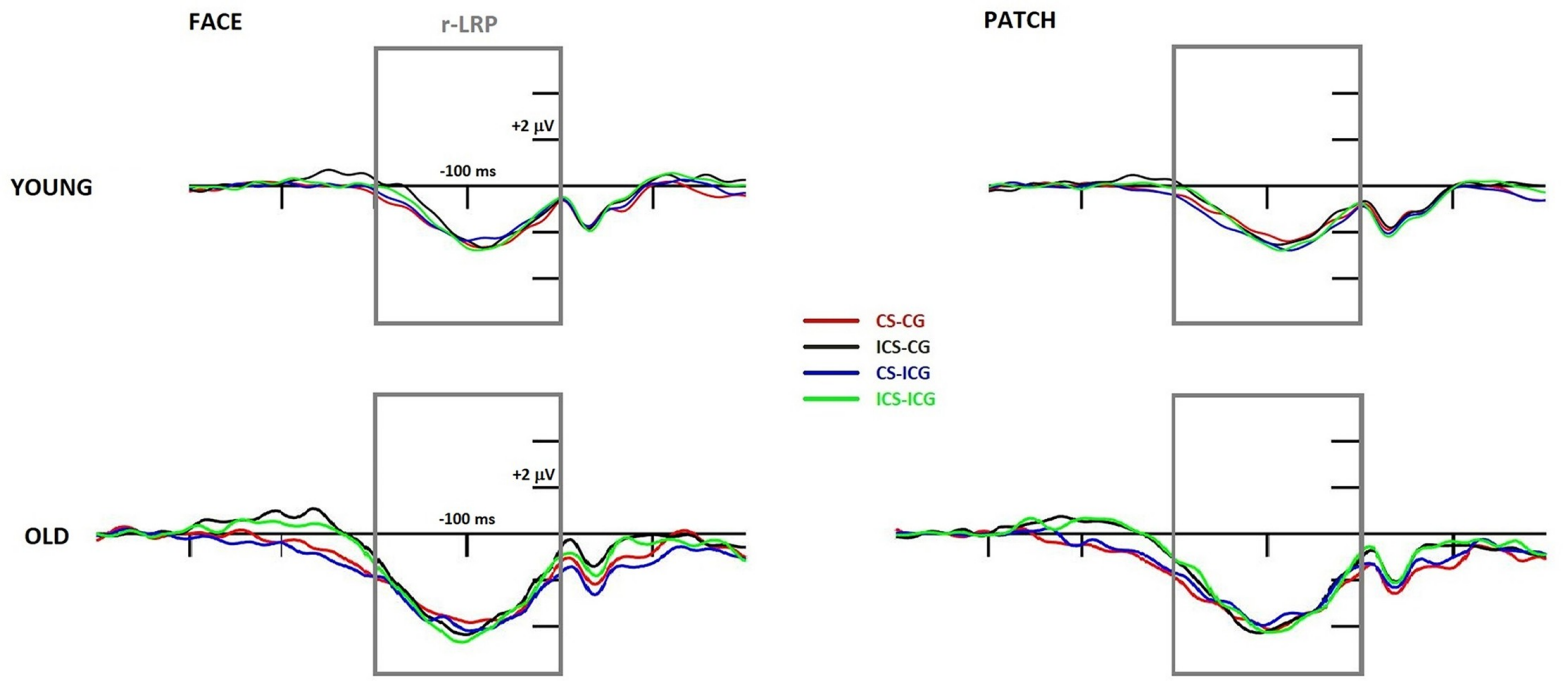
Response-locked LRP component at the C3-C4 electrode difference (contra-minus-ipsilateral to the responding hand) in young (top panel) and older (bottom panel) adults and in face (left side) and patch (right side) trials. The x-axis represents the epoch from -400 ms to 200 ms around the time-point of the response. CS-CG (congruent Simon, congruent gaze) waveforms are displayed in red, ICS (incongruent Simon)-CG waveforms in black, CS-ICG (incongruent gaze) waveforms in blue, and ICS-ICG waveforms in green. Grey rectangles represent the search window for maximal r-LRP amplitude in every subject.

*Response-locked LRP*. As shown in Fig 8, older adults had a significantly larger (*Age* main effect for amplitude: F(1,43) = 8.31, p = 0.006, η_p_^2^ = 0.16) and earlier (*Age* main effect for peak latency: F(1,43) = 5.78, p = 0.021, η_p_^2^ = 0.12) r-LRP component than young adults. Additionally, older participants had significantly earlier r-LRP onset as well (*Age* main effect: F(1,43) = 22.35, p < 0.001, η_p_^2^ = 0.34), and this effect was significant only after face cues, not after patches (*Cue* × *Age* interaction: F(1,43) = 9.35, p = 0.004, η_p_^2^ = 0.18).

## Discussion

Our purpose was not only to investigate the age-related changes of different cognitive control processes in a gaze cued Simon task, but also to compensate for the age-related decline in target stimulus and response-conflict evaluation, as well as motor response inhibition in older adults. We hypothesized that older adults, as opposed to the young adults, cannot ignore the task-irrelevant faces, so therefore their gaze would enhance target processing and hence the Simon effect in the congruent gaze cueing condition: the facilitation effect of gaze cueing would decrease the reaction time in the older group in the Simon congruent condition, whilst having an inhibitory effect would increase it in the incongruent Simon trials.

In the Simon task we replicated the earlier results found by others (e.g., [19, 41,42]) as: (1) there were less errors and (2) responses were faster in congruent than in incongruent trials; (3) P3b latency was longer in the incongruent Simon condition; (4) the amplitude was smaller when compared with the congruent Simon condition; (5) initial wrong side activation started in incongruent but not in congruent Simon condition; and as a consequence, (6) s-LRP latency was longer in incongruent compared to congruent Simon condition. From these findings we can conclude two things: firstly, when compared to the congruent trials, the incongruent ones had a higher task demand and consequently consumed more cognitive resources [22]. And secondly, there was an increased interference in the response selection stage in the incongruent condition [43,44].

Although in a recent study by Rey-Mermet and Gade [45] the general inhibitory deficit in older age was questioned, they admitted that in some types of task, such as the Simon task, more results were necessary to draw a final conclusion. Concerning age differences, we found a typical pattern of slower responses for older people in their reaction times [46], slowed attentional processing indexed by the N2pc latency [19], and also a delayed P3b component [47], but N2pc amplitude data did not confirm they had a less efficient stimulus discrimination [19]. Later s-LRP onset and a longer latency in older adults, compared with young participants, showed slower target processing within the visuomotor circuits, while earlier r-LRP onset and its longer latency indicated the prolongation of motor processing and movement preparation in older compared to young adults [48].The results of this study confirmed that the elderly, when compared with the young group, not only had their inhibitory functions impaired; but also had an increased response activation threshold. This was evident by the larger Simon effect (reaction time), and the larger amplitude of the s-LRP and r-LRP components [29, 31]. Also, larger amplitude of the positive dip, indexing enhanced activation of the wrong response showed that older adults were less effective in inhibiting their tendency to react according to the side of the target stimulus. The latency difference between the positive and negative s-LRP component in the incongruent Simon condition also indicated that older participants needed more time to inhibit the wrong side activation and reverse motor activation to reach the correct response. Despite the older group having a slower reaction time they made significantly less errors in the Simon task than the younger group, which suggests that they could evaluate cognitive conflict more effectively. However, we might put this down to the fact that they had higher IQ scores, and their cognitive control and conflict resolving processes were probably unimpaired, and they applied reactive control to resolve cognitive conflicts [49,50]. Another explanation could be their speed-accuracy trade-off [51], namely the inverse relation between speed and accuracy in determining task resolution.

Our aim was to find out how gaze influences the Simon effect in different age-groups. To our knowledge there has not been any examination of responses to gaze-cueing in the elderly. Although Frischen et al. [2] suggest in their review that biologically relevant and irrelevant central cues cause similar attention shifts, our reaction time data showed that the measured modifications were the result of the gaze being a social stimulus, rather than merely due to the movement of the two dots: the patch, unlike the facial stimuli, did not evoke any gaze cueing effect. It is of note that the gaze was an unattended stimulus in our experiment by instruction, and participants had to ignore this information. If the gaze influenced the results, that shows they were not able to inhibit their tendency to pay attention to the face or the patch. And according to the inhibition deficit theory [1] we assumed that older people would be more affected by the gaze.

We hypothesized that if participants could not ignore the face, then its gaze would orient their attention and facilitated target processing [8]; and consequently, the congruent gaze would have had a facilitating effect in congruent but an inhibitory effect in incongruent Simon conditions when compared to incongruent gaze effects. We found there to be differences in the two gaze conditions in both behavioural (RT, errors) and ERP data (N2pc, wrong-side activation of the s-LRP). The reaction time was longer in the congruent trials when the gaze focused on the target stimulus, which was true for faces but not for patches, indicating the importance of the face as a social stimulus and also that the gaze influenced the focus of attention. The larger N2pc amplitude in congruent compared to incongruent gaze condition also showed the increased visuospatial attention when the gaze focused on the target. The positive dip of s-LRP wrong-side activation in incongruent Simon condition showed a larger amplitude in congruent rather than incongruent gaze condition, which shows the expected extra inhibitory effect of the gaze.

The age-groups differed in three indexes. Firstly, while the number of errors was not influenced by the gaze in the young group, this being the group which we hypothesized could effectively inhibit the task-irrelevant information; the older group had more errors in the congruent rather than incongruent gaze condition when the Simon task was incongruent, but not when it was congruent. Which we had interpreted as they being not able to exclude the interference of the gaze, which caused a further demand on their task solving ability. Secondly, this result was also confirmed by the reaction time data which was longer in the incongruent Simon condition when the face focused on the target stimulus compared to the incongruent gaze condition in older adults. This was not observed in the young group who could ignore the irrelevant stimuli more effectively. Thirdly, the r-LRP onset also showed that the socially important but task-irrelevant face, but not the patch, resulted in a prolongation of the motor processing and response preparation in the older group.

Our results partly contradict some previous data. As far as we know, there is only one paper [17] in which gaze cueing was used to modify the Simon effect, and opposite results were found in the incongruent Simon condition, namely reaction time was longer when the gaze was incongruent than congruent. Also, Wascher and Wolber [52] and Proctor et al. [53] found that the Simon effect was enhanced if a pre-cue gave advance information about the likely response side, but it was not affected if the pre-cue indicated the likely location of the target stimulus, while our N2pc data suggests that visuospatial attention increased when the gaze focused on the target stimulus and not the response side. In these previous experiments only young adults were tested, the pre-cue (a schematic face, hand or arrow) was an attended stimulus and also an informative cue in the latter cases, while in our experiment older adults were affected by the direction of the uninformative gazes, which were task-irrelevant real faces, varying between trials. These parameters might be responsible for the different outcomes, but this deserves further study.

In summary, earlier results in connection with the effect of aging on response conflict evaluation have been confirmed by our results; specifically aging and stimulus-response incongruency delay target stimulus processing within the visuomotor circuits. All in all, motor response inhibition and response conflict evaluation becomes less effective with aging and gaze cueing has a modifying effect principally on older adults.

## Conclusions

Our results confirmed in older adults their decreased efficiency in inhibitory processes as well as their interference-sensitivity [1]. In the visuospatial domain the older age-group was significantly slower than the young one. Moreover, aging and stimulus-response incongruency delayed the evaluation of response and decreased the efficiency of motor inhibition in the Simon task. However, by having sufficient time, older adults could solve the tasks correctly. Although gaze-cueing could modify task processing, the gaze did not have a facilitatory effect that could compensate for age-related differences, but rather drew attention from the side of the response and added a further burden on cognitive processing.
